# The effect of dicarbonyl stress on the development of kidney dysfunction in metabolic syndrome – a transcriptomic and proteomic approach

**DOI:** 10.1186/s12986-019-0376-1

**Published:** 2019-08-01

**Authors:** Irena Markova, Martina Hüttl, Olena Oliyarnyk, Tereza Kacerova, Martin Haluzik, Petr Kacer, Ondrej Seda, Hana Malinska

**Affiliations:** 1 Centre for Experimental Medicine, Institute for Clinical and Experimental Medicine, Prague, Czech Republic; 2 Department of Chemistry, University College London, London, UK; 3 Czech University of Life Sciences, Prague, Czech Republic; 4 Institute of Biology and Medical Genetics, First Faculty of Medicine, Charles University & General University Hospital in Prague, Prague, Czech Republic

**Keywords:** Metabolic syndrome, Methylglyoxal, Kidney dysfunction, Transcriptomics, Proteomics, Metabolomics, Microvascular complications

## Abstract

**Background and aims:**

Dicarbonyl stress plays an important role in the pathogenesis of microvascular complications that precede the formation of advanced glycation end products, and contributes to the development of renal dysfunction. In renal cells, toxic metabolites like methylglyoxal lead to mitochondrial dysfunction and protein structure modifications.

In our study, we investigated the effect of methylglyoxal on metabolic, transcriptomic, metabolomic and proteomic profiles in the context of the development of kidney impairment in the model of metabolic syndrome.

**Materials and methods:**

Dicarbonyl stress was induced by intragastric administration of methylglyoxal (0.5 mg/kg bw for 4 weeks) in a strain of hereditary hypertriglyceridaemic rats with insulin resistance and fatty liver.

**Results:**

Methylglyoxal administration aggravated glucose intolerance (AUC_0–120_ *p* < 0.05), and increased plasma glucose (*p* < 0.01) and insulin (*p* < 0.05). Compared to controls, methylglyoxal-treated rats exhibited microalbuminuria (*p* < 0.01). Targeted proteomic analysis revealed increases in urinary secretion of pro-inflammatory parameters (MCP-1, IL-6, IL-8), specific collagen IV fragments and extracellular matrix proteins. Urine metabolomic biomarkers in methylglyoxal-treated rats were mainly associated with impairment of membrane phospholipids (8-isoprostane, 4-hydroxynonenal).

Decreased levels of glutathione (*p* < 0.01) together with diminished activity of glutathione-dependent antioxidant enzymes contributed to oxidative and dicarbonyl stress. Methylglyoxal administration elevated glyoxalase 1 expression (*p* < 0.05), involved in methylglyoxal degradation. Based on comparative transcriptomic analysis of the kidney cortex, 96 genes were identified as differentially expressed (FDR < 0.05). Network analysis revealed an over-representation of genes related to oxidative stress and pro-inflammatory signalling pathways as well as an inhibition of angiogenesis suggesting its contribution to renal fibrosis.

**Conclusion:**

Our results support the hypothesis that dicarbonyl stress plays a key role in renal microvascular complications. At the transcriptome level, methylglyoxal activated oxidative and pro-inflammatory pathways and inhibited angiogenesis. These effects were further supported by the results of urinary proteomic and metabolomic analyses.

## Introduction

Diabetic nephropathy (diabetic kidney disease) is a major chronic microvascular complication of diabetes which is characterised by a progressive increase in albuminuria and a decline in the glomerular filtration rate. Although hyperglycaemia represents an important risk factor in the development of microvascular complications in the kidney, intensive glycaemic control as a sole therapeutic solution is insufficient to prevent these complications [1].

According to recent studies, dicarbonyl stress plays an important role in the pathogenesis of microvascular renal complications that precede the formation of advanced glycation end products (AGEs) [2].

Dicarbonyl stress occurs as a consequence of the imbalance between the generation of reactive dicarbonyls (like methylglyoxal) and their metabolism via the glyoxalase system. The accumulation of dicarbonyls and impaired glyoxalase 1 function may be involved in complications of vascular dysfunction, especially at a microvascular level, and both are associated with microvascular complications in the kidney.

The recently published clinical study ADDITION-DK [3] supports the role of methylglyoxal in the pathogenesis of renal microvascular complications and methylglyoxal (MGO) was found to be associated with detrimental changes in kidney function in individuals with type 2 diabetes (T2D).

Excessive generation of dicarbonyls activates inflammatory processes, increases oxidative stress, impairs glucose tolerance, and leads to AGE production. In a study of American Indians with type 2 diabetes, AGEs derived from MGO (carboxyethyl lysine and methylglyoxal hydroimidazolone) improved the accuracy of predicting renal function loss over traditional renal risk factors and correlated with the severity of diabetic kidney disease [4].

In renal cells, MGO directly inhibits the electron respiratory chain (leading to mitochondrial dysfunction) and modifies protein structure which in turn may affect their function. Indirectly, MGO can also disturb different signalling pathways associated with vascular complications [5]. However, the exact pathophysiological mechanisms underlying the detrimental effects of MGO on the kidney remain elusive.

Assessing global gene expression changes in the kidney and identifying urine proteomic and metabolomic biomarkers can help to elucidate the development of these complications and determine early risk markers.

In this study, we investigated the effect of MGO on metabolic, transcriptomic and proteomic profiles in the context of the development of kidney impairment using an experimental model of metabolic syndrome, the hereditary hypertriglyceridaemic rat (HHTg). Originating from Wistar rats, this strain exhibits dyslipidaemia, insulin resistance, hyperinsulinaemia, fatty liver, oxidative stress and low-grade chronic inflammation, and is accepted model of metabolic syndrome and prediabetes [6].

## Methods

### Animals and diet

All experiments were performed in agreement with the Animal Protection Law of the Czech Republic (311/1997), which is in compliance with European Community Council recommendations (86/609/ECC) for the use of laboratory animals, and approved by the Ethics Committee of the Institute for Clinical and Experimental Medicine, Prague.

Male HHTg rats were fed a standard chow diet (23% proteins, 43% starch, 7% fat, 5% fibre and 1% vitamin and mineral mixture, Bonagro, Czech Republic) and were maintained under temperature (22 °C) and humidity-controlled conditions at a 12 h/12 h light-dark cycle. At all times, the animals had free access to food and water. The rats (5 months old) were divided into 2 group (8 animals in each group): MGO-treated group of HHTg rats - MGO (Sigma) was administered intragastrically 3 times a week at a dose of 0.5 mg/kg body weight (BW) for 4 weeks. In the control group, rats were intragastrically administered water for 4 weeks. At the beginning of the study, there were no differences in body weight, serum glucose and triglycerides between both groups of HHTg rats. At the end of experiment, animals were sacrificed by decapitation in a postprandial state. Aliquots of serum and tissue samples were collected at − 80 °C for analyses.

### Analytic methods & biochemical analysis

Serum levels of triglycerides, glucose, total cholesterol and FFA were measured using commercially available kits (Erba Lachema, Czech Republic & Roche Diagnostics, Germany). Serum insulin and adiponectin concentrations were determined using the Rat Insulin ELISA kit (Mercodia AB, Sweden) and the Rat HMW Adiponectin ELISA kit (MyBioSource, USA), respectively.

For the oral glucose tolerance test (OGTT), blood glucose was determined after a glucose load (3 g of glucose/kg BW) administered intragastrically after overnight fasting. Blood glucose concentrations were determined by analysing blood samples collected from the tail at 0, 30, 60, and 120 min after glucose loading. The area under the glycaemic curve (AUC) was calculated over a 120-min period.

For the determination of triglycerides in tissues, samples were extracted in chloroform/methanol. The resulting pellet was dissolved in isopropyl alcohol, with triglyceride content determined by an enzymatic assay (Erba Lachema, Czech Republic).

The levels of reduced (GSH) and oxidised (GSSG) forms of glutathione were determined by high-performance liquid chromatography with fluorescent detection using a HPLC diagnostic kit (Chromsystems, Germany).

Antioxidant enzyme activities were measured using commercially available kits (Sigma-Aldrich & Cayman Chemicals).

The level of albumin in urine was analysed using a high-performance liquid chromatography method with UV-VIS detection according to Contois et al. [7] and adjusted for creatinine concentration. Creatinine concentration was determined by enzymatic creatinine assay kit (Roche Diagnostics).

### Dicarbonyl stress parameters

Dicarbonyl concentrations were determined after derivatisation with 1,2-diaminobenzene using a HPLC method with fluorescence detection according to Fleming and Bierhaus [8].

GLO1 activity was analysed using a method described by Arai [9]. Red blood cells were collected by centrifugation of blood (EDTA) samples and washed 3 times with 0.01 M PBS (pH 7.4). Washed cells were lysed using cold deionised water. Haemoglobin concentrations were determined according to Drabkin’s assay (Sigma).

### Transcriptome profiling and relative expression

Transcriptome assessment of the kidney cortex was performed using the GeneChip® Rat Gene 2.1 ST Array Strip on the Affymetrix Gene Atlas System (Thermo Fisher Scientific, Waltham, MA, USA). Total RNA was extracted from the kidney using phenol-chloroform and purified using the RNeasy Mini kit (Qiagen, Valencia, USA). The quality and integrity of the total RNA were evaluated on the Agilent 2100 Bioanalyzer system (Agilent, Palo Alto, CA). Only samples surpassing the minimum quality threshold (RIN > 8.0) were used for subsequent transcriptome assessment (*n* = 8 in each group). The whole procedure, including several phases of reverse transcription, was performed according to the protocol recommended by the manufacturer. The microarray data were deposited in ArrayExpress database (www.ebi.ac.uk/arrayexpress) under accession number E-MTAB-7690.

Microarray results were validated by qPCR. Reverse transcription and quantitative real-time PCR analysis was performed using the TaqMan RNA-to-C_T_ 1-Step Kit, TaqMan Gene Expression Assay (Applied Biosystems, USA), and the ViiA™ 7 Real Time PCR System (Applied Biosystems, USA). Relative expression was determined after normalisation against β-actin as an internal reference and calculated using the 2^-ΔΔCt^ method.

### Proteomic and metabolomic markers in urine

Urine markers of oxidative stress were analysed using a liquid chromatography/mass spectrometry system (triple quadrupole mass spectrometer with electrospray ionisation). To implement multimarker screening, we performed two types of analysis: the first to detect compounds containing amino groups and the second to detect compounds with aldehyde and carboxylic groups [10].

Urine protein candidate biomarkers were analysed using MALDI-TOF MS by prior immunomagnetic isolation. Custom-designed antibody microarrays were used to confirm MS data by comprising the relative content of different analytes in the samples.

### Statistical and pathway analysis

All data were statistically evaluated using the unpaired Student’s t-test, while categorical variables were analysed using Fisher’s exact test (used statistical software GraphPad InStat 3.1). Statistical significance was defined as *P*<0.05, with data expressed as mean ± SEM.

For transcriptome data, hybridisation and quality control were evaluated using the Affymetrix Expression Console (Thermo Fisher Scientific, Waltham, MA, USA). Data were then normalised (Robust Multi-array Average, RMA); gene expressions were compared between the MG-treated group and the control group using analysis of variance with multiple comparison adjustment and the false discovery rate method (FDR < 0.05), as implemented in PARTEK Genomics Suite 6.6 (Partek Inc., St. Louis, MI, USA). Transcripts that were significantly differentially expressed by more than 1.2 fold between both groups (FDR<0.1) were processed for gene enrichment and network/pathway analysis using Ingenuity Pathway Analysis software (Qiagen Redwood City, Inc., Redwood City, CA, USA). Proteomic and metabolomic biomarkers were calculated using XLSTAT software (https://www.xlstat.com).

## Results

### Effect of methylglyoxal on metabolic parameters

During the study, no differences in food and drink consumption between the experimental groups of rats were observed. Although MGO administration did not alter body weight, it aggravated glucose intolerance (AUC_0–120_) and increased fasting plasma glucose and insulin (Table 1). MGO-treated rats exhibited significant changes in serum lipids, while serum triglycerides were reduced and total serum cholesterol and HDL-cholesterol were significantly elevated compared to controls. However, triglyceride concentrations in tissues were comparable in both groups. MGO administration increased MGO concentrations in serum, in the kidney and liver (Table 1).

**Table 1:** Metabolic parameters in serum and tissues in HHTg rats and after methylglyoxal administration (HHTg + MGO)

	HHTg	HHTg + MGO	*P*<
Body weight (g)	412 ± 3	397 ± 6	n.s.
Glucose (mmol/l)	7.1 ± 0.2	8.8 ± 0.2	0.001
Insulin (μmol/l)	0.25 ± 0.03	0.52 ± 0.03	0.05
AUC_0–120_ mmol/l	930 ± 26	1006 ± 18	0.05
FFA (mmol/l)	0.43 ± 0.05	0.49 ± 0.06	n.s.
Adiponectin (μg/ml)	2.90 ± 0.34	2.78 ± 0.51	n.s.
Serum triglycerides (mmol/l)	4.80 ± 0.49	3.21 ± 0.51	0.05
Serum cholesterol (mmol/l)	1.19 ± 0.07	1.56 ± 0.99	0.05
HDL-C (mmol/l)	0.59 ± 0.03	1.06 ± 0.14	0.05
Triglycerides in the liver (μmol/g)	13.01 ± 1.58	13.02 ± 0.94	n.s.
Triglycerides in the kidney (μmol/g)	8.55 ± 1.61	6.81 ± 1.16	n.s.
Methylglyoxal in serum (nmol/ml)	0.24 ± 0.02	0.37 ± 0.02	0.01
Methylglyoxal in the kidney (nmol/mg)	0.59 ± 0.08	0.86 ± 0.09	0.01
Methylglyoxal in the liver (nmol/mg)	1.62 ± 0.12	5.26 ± 0.58	0.001

Data are mean ± SEM; *n* = 8

### Effect of methylglyoxal on parameters of oxidative and dicarbonyl stress in the kidney

**Fig. 1:**
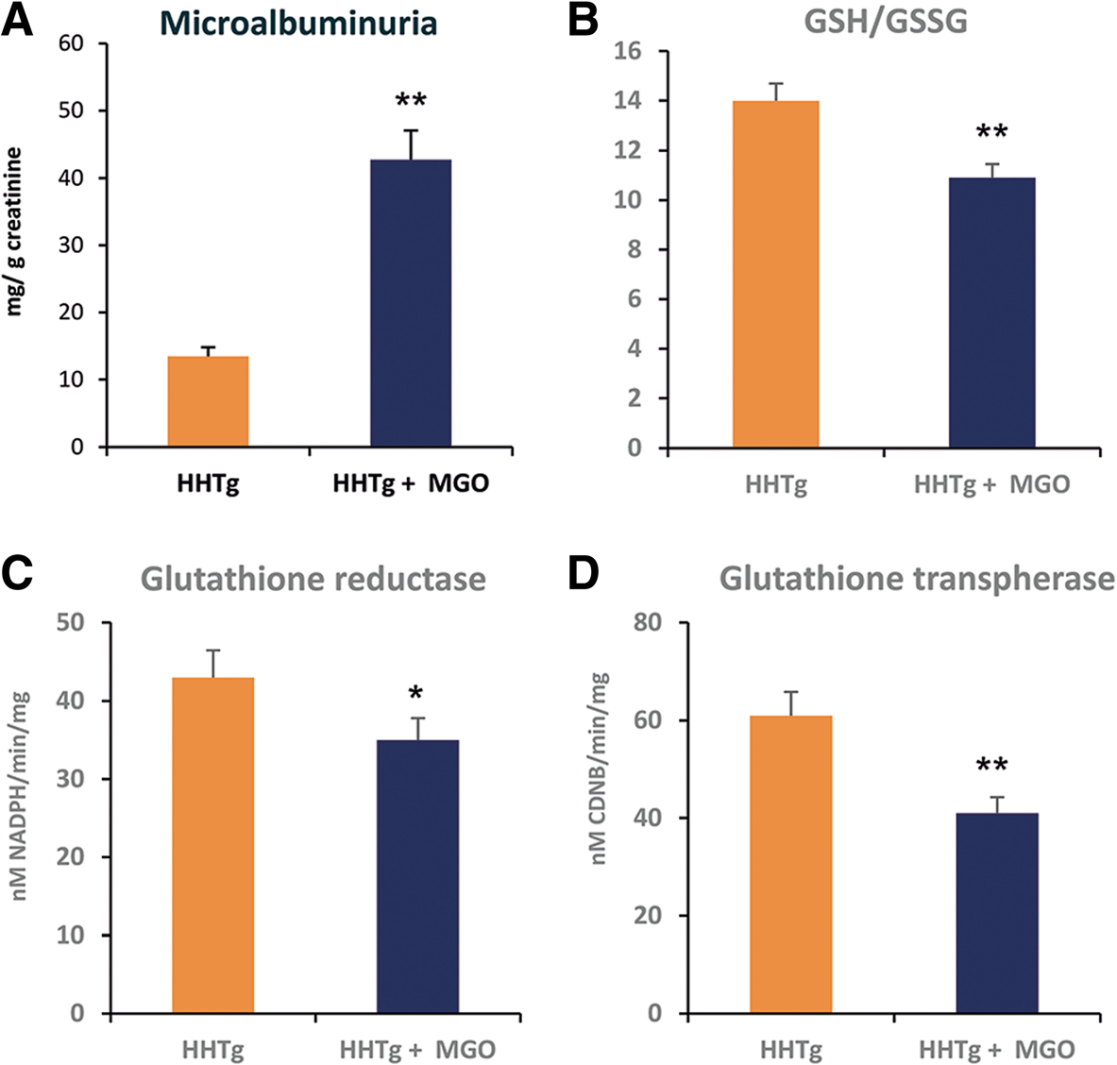
Effect of MGO treatment on urinary albumin (Panel **a**) and oxidative stress parameters in the kidney. Ratio of reduced to oxidised glutathione (Panel **b**); glutathione reductase activity (Panel **c**) and glutathione transferase activity (Panel **d**). Data (panels **a-d**) are measured in doublets using *n* = 8 rats per group per analyses. Data are expressed as means (SEM) and analysed by two-tailed unpaired Student’s *t* test. * *p* < 0.05, ** *p* < 0.01, *** *p* < 0.001

**Fig. 2:**
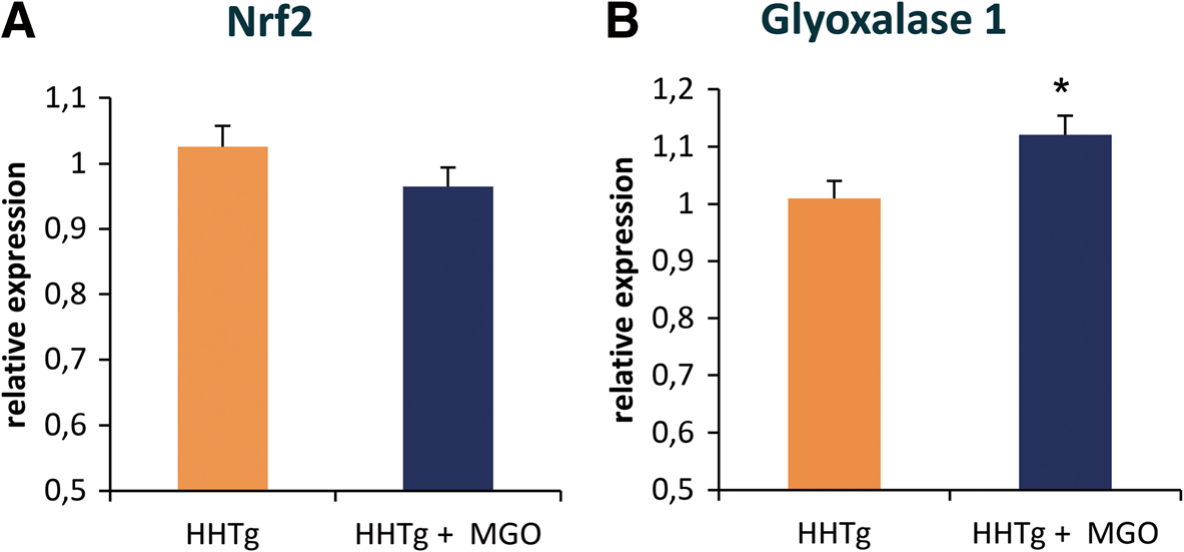
Effect of MGO treatment on relative expression of nuclear factor NRF2 and the GLO1 enzyme in the kidney. Relative expression of nuclear factor, erythroid 2 like 2 (NRF2, Panel **a**) and glyoxalase 1 (GLO1, Panel **b**) in the kidneys of MGO-treated vs. control male hereditary hypertriglyceridaemic rats (HHTg). Data (panels **a-b**) are measured in triplets using *n* = 8 rats per group per analyses. Data are expressed as means (SEM) and analysed by two-tailed unpaired Student’s *t* test. * *p* < 0.05, ** *p* < 0.01, *** *p* < 0.001

In MGO-treated rats, microalbuminuria was associated with decreased levels of reduced glutathione in the kidney cortex together with significantly decreased activity of glutathione-dependent antioxidant enzymes (glutathione reductase and glutathione transferase) (Fig. 1). However, relative expression of transcriptional factor NRF2, which is involved in the antioxidant response, did not change (Fig. 2). MGO administration elevated gene expression of glyoxalase 1 (Fig. 2), which is involved in MGO degradation. There were no differences in glyoxalase 1 activity between both groups (data not shown).

### Effect of methylglyoxal on the kidney transcriptome

Based on comparative transcriptomic analysis of the kidney cortex, 96 genes were identified as differentially expressed (FDR < 0.05) in methylglyoxal-treated rats compared to controls, with 54 relatively upregulated and 42 downregulated transcripts. Among the top upregulated genes in MGO-treated rats were nuclear receptor subfamily 1, group D, member 1 (NR1D1), insulin-like growth factor-binding protein 1 (IGFBP1) and D site of albumin promoter (albumin D-box)-binding protein (DBP). The top genes downregulated by MGO included ADAM metallopeptidase with thrombospondin type 1 motif, 1 (ADAMTS1), collagen, type VI, alpha 2 (COL6A2), and sterol regulatory element-binding transcription factor 1 (SREBF1). The complete set of differentially expressed transcripts is provided in ESM Table 1.

**Fig. 3:**
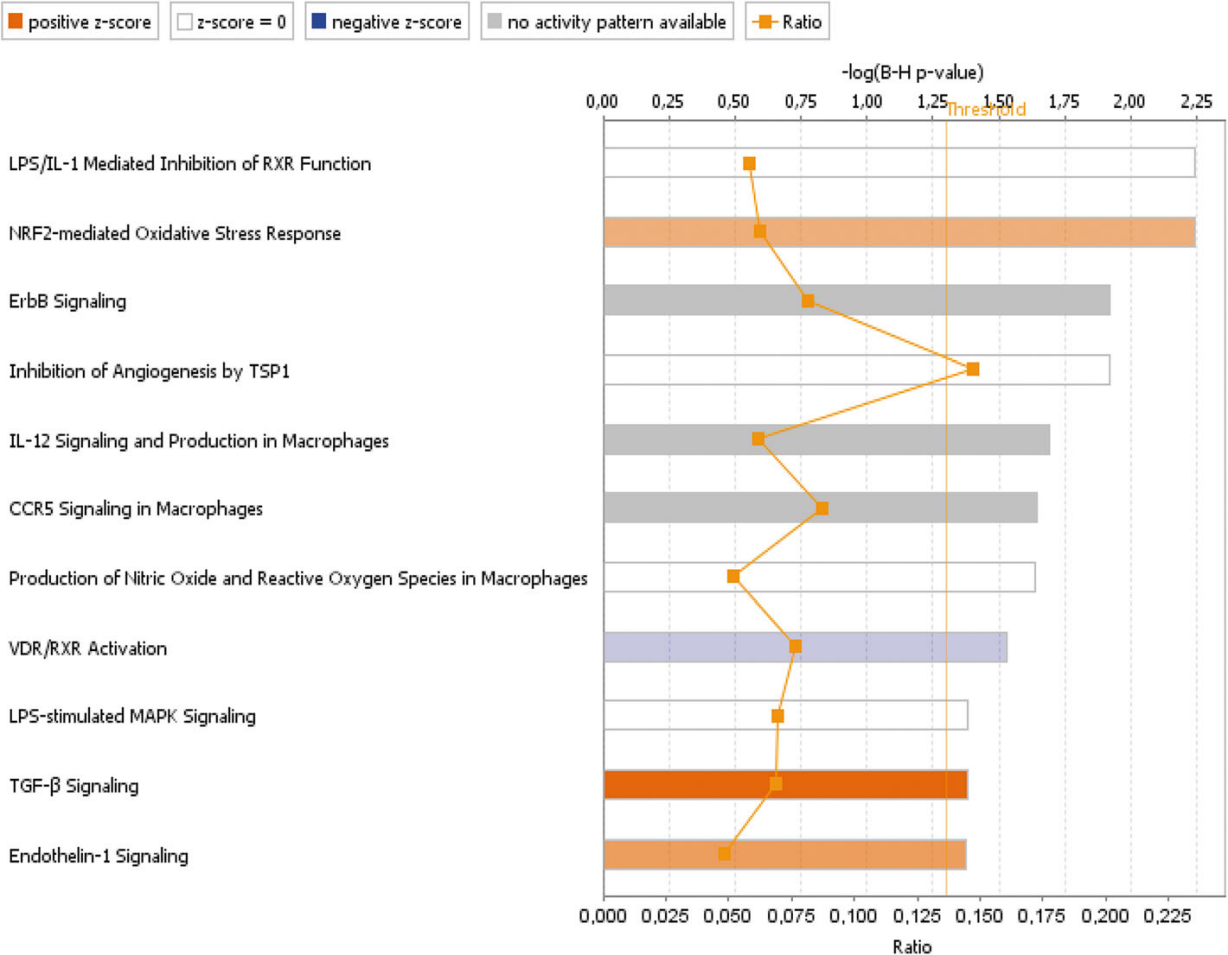
Significantly enriched canonical pathways in MGO-treated rats. Canonical pathways were identified using Ingenuity Pathway Analysis (IPA, Qiagen Redwood City, Inc., Redwood City, CA, USA). Bars indicate significance levels of individual pathways (scored as –log (*p*-value) based on Fisher’s exact test, upper x-axis); “threshold” (yellow line) indicates the Benjamini-Hochberg-corrected minimum significance level. Ratio (square markers connected by line, lower x-axis) refers to the number of genes from the dataset that map to the shown pathway divided by the total number of molecules that define the canonical pathway within the IPA knowledge base. Shades of orange and blue are proportional to values of positive or negative z-scores and used for evaluating the overall activation or inhibition of a given pathway

Using all differentially expressed genes, we systematically assessed their enrichment (in ontological categories or disease-related gene sets) and potential functional connections. As shown in Fig. 3, significantly over-represented canonical pathways reveal major activation of the TGF-β signalling pathway (*p*-value = 2.22E-03), a hallmark of renal fibrosis. Other significantly enriched functional categories in the kidney after methylglyoxal were related to oxidative stress (NRF2-mediated signalling pathway, *p*-value = 4.58E-05), inflammation (LPS/IL-1-mediated pathway, *p*-value = 4.15E-05) and inhibition of angiogenesis (thrombospondin-1 signalling pathway, *p*-value = 1.59E-04).

Next, we aimed to identify upstream regulators potentially responsible for the MGO-induced transcriptomic shift and predict their activation or inhibition based on gene expression changes in MGO-treated and control rats. Using an algorithm based on the expected causal effects between upstream regulators and their targets implemented in the Ingenuity Pathway Analysis software [11], five entities were predicted to be activated: acyl-CoA oxidase 1 (ACOX1, z-score 2.14, Fisher’s exact *p* = 1.76E-03) through its involvement in fatty acid oxidation and inflammation, LIM homeobox protein 1 (LHX1, z-score 2.43, *p* = 5.40E-03) as a participant in the proliferative response to kidney injury, cystic fibrosis transmembrane conductance regulator (CFTR, z-score 2.24, *p* = 9.06E-03), hepatocyte nuclear factor 1α (HNF1A, z-score 2.90, *p* = 9.28E-03), and X-box binding protein-1 activated by endoplasmic reticulum stress (XBP1, z-score 2.24, *p* = 1.62E-02). In contrast, only two regulators were predicted to be significantly inhibited: insulin 1 (INS1, z-score − 2.62, *p* = 2.34E-02) and enhancer of zeste 2 polycomb repressive complex 2 subunit (EZH2, z-score − 2.22, *p* = 4.84E-02). Analysis of toxicity functions and toxicity lists, which link gene expression differences to clinical pathology endpoints, revealed particular enrichment in categories associated with the long-term renal injury pro-oxidative response panel and downregulation of genes involved in recovery from ischaemic acute renal failure (ESM Fig. 1). Network analysis revealed over-representation of genes participating in oxidative stress and pro-inflammatory signalling pathways, as well as inhibition of angiogenesis. Together, these data indicate the involvement of MGO in renal fibrosis and the early phase of renal failure, which is supported by the overall gene expression pattern we observed in upstream regulator-based networks.

### Effect of methylglyoxal on urinary parameters

Compared to controls, MGO-treated rats exhibited significantly more pronounced microalbuminuria (Fig. 1) and higher urine lactate levels (12.87 ± 0.90 vs 6.13 ± 0.15 mmol/ml, *p* < 0.05). Untargeted proteomic analysis identified 509 proteins, peptides and fragments (data not shown). As shown in Table 2, targeted proteomic analysis revealed increases in urinary secretion of pro-inflammatory parameters (MCP-1, IL-6, IL-8), specific collagen IV fragments (endostatin), α1-antitrypsin and extracellular matrix protein (heparan sulphate). However, urinary secretion of endothelial growth factor (EGF) significantly reduced after MGO administration. Urine metabolomic biomarkers in MGO-treated rats were mainly associated with impairment of membrane phospholipids (MDA, 8-isoprostane, 4-hydroxynonenal). Metabolomic impairment parameters of DNA (8-hydroxyguanosin), RNA (5-hydroxymethyluracyl), proteins and inflammatory parameters (leukotriene D4) were also identified in urine (Table 2). Altogether, 33 tested parameters of inflammation and oxidative stress were identified; only significantly changed markers are shown in Table 2.

**Table 2:** Proteomic and metabolomic markers in urine

	HHTg	HHTg + MGO	*P*<
Proteomic markers			
IL-6 (pg/ml)	44.33 ± 2.31	91.43 ± 5.88	0.001
IL-8 (pg/ml)	18.33 ± 0.29	49.43 ± 1.60	0.001
MCP-1 (ng/ml)	1.57 ± 0.02	3.76 ± 0.21	0.001
EGF _(_ng/ml)	4.95 ± 0.17	2.27 ± 0.01	0.001
α-1 antitrypsin (ng/ml)	13.90 ± 2.89	24.47 ± 2.11	0.001
IgA-uromodulin (ng/ml)	60.50 ± 5.20	68.71 ± 0.80	0.05
Tumstatin (pg/ml)	79.33 ± 1.15	68.00 ± 5.61	n.s.
Endostatin (pg/ml)	71.50 ± 4.04	102.14 ± 5.08	0.01
Heparan sulphate (μg/ml)	0.09 ± 0.01	0.31 ± 0.01	0.05
Metabolomic markers			
Malondialdehyde (ng/ml)	22.25 ± 0.61	31.40 ± 0.32	0.001
4-hydroxyhexenal (ng/ml)	14.77 ± 0.43	23.71 ± 0.76	0.001
4-hydroxynonenal (ng/ml)	23.93 ± 0.12	32.72 ± 0.55	0.001
Hexanal _(_ng/ml)	15.82 ± 0.52	20.30 ± 0.56	0.01
Heptanal (ng/ml)	22.68 ± 0.20	28.62 ± 1.12	0.01
Octanal (ng/ml)	10.07 ± 0.09	11.87 ± 0.40	0.001
Nonanal (ng/ml)	12.20 ± 0.17	14.96 ± 0.07	0.001
Decanal (ng/ml)	9.35 ± 0.23	11.34 ± 0.32	0.001
Dodecanal (ng/ml)	6.77 ± 0.35	7.39 ± 0.32	n.s.
8-isoprostane (pg/ml)	21.33 ± 0.29	29.09 ± 3.53	0.01
3-nitrotyrosine (pg/ml)	49.67 ± 0.01	71.60 ± 0.53	0.001
o-nitrotyrosine (pg/ml)	57.17 ± 1.15	77.36 ± 1.49	0.001
3-chlorotyrosine (pg/ml)	22.67 ± 2.02	35.54 ± 1.60	0.01
8-hydroxyguanosine (pg/ml)	198.00 ± 4.91	231.54 ± 3.69	0.05
5-hydroxymethyluracil (pg/ml)	93.00 ± 2.02	115.07 ± 13.76	0.05
Leukotriene B_4_ (pg/ml)	48.17 ± 8.66	81.86 ± 28.86	n.s.
Leukotriene C_4_ (pg/ml)	64.17 ± 4.91	66.67 ± 0.53	n.s.
Leukotriene D_4_ (pg/ml)	53.83 ± 1.73	60.69 ± 1.64	0.05
Leukotriene E_4_ (pg/ml)	119.17 ± 5.77	120.72 ± 0.12	n.s.

Data are shown as mean ± SEM; *n* = 8/group

## Discussion

MGO represents an important pathogenic factor in the development of renal microvascular complications in T2D that precede AGE formation and the onset of hyperglycaemia. We previously reported [12] that dyslipidaemia in hypertriglyceridaemic rats leads to elevated levels of MGO and MGO-derived AGEs in serum and tissues. Blood levels of AGEs, especially those derived from MGO, predict renal function loss and correlates with T2D in American Indians [4]. AGEs accumulate in glomeruli, where they increase gene expression of extracellular matrix type IV collagen and induce cross-linked protein formations.

MGO administration in our study has been shown to induce severe dicarbonyl stress comparable to levels in T2D patients with poorly controlled diabetes. In our study, MGO aggravated glucose intolerance and increased insulin resistance in the tissues of hypertriglyceridaemic rats, corroborating previous findings [13]. Although MGO administration had no effect on ectopic lipid deposition or body weight in the current study, MGO-treated HHTg rats exhibited changes in blood lipid profiles as serum triglycerides were decreased and total serum cholesterol was significantly increased. Similar findings have been reported in other experimental studies [14] and may be associated with the effect of MGO on the activity of lipoprotein lipase rather than its influence on lipolysis.

The changes we observed were accompanied by downregulation of sterol regulatory element-binding transcription factor 1 (SREBF1) and upregulation of the elongase of very long chain fatty acids 2 (ELOVL2), an enzyme involved in fatty acid synthesis, storage and oxidation [15].

In our study in hypertriglyceridaemic rats, MGO aggravated oxidative stress in the kidney, glutathione and glutathione-dependent antioxidant enzymes were significantly reduced. However, glutathione depletion in the kidney had no effect on GLO1 activity. Elevated relative expression of GLO1 in the kidney likely serves as a compensatory mechanism that protects renal cells against the glycation by MGO and helps to sustain unchanged GLO1 activity in the kidney. Similar results with GLO1 expression have been observed in db/db diabetic mice [16]. GLO1 ensures MGO detoxification and that its expression remains under the control of transcription factor NRF2, which orchestrates the antioxidant response. Comparative expression analysis of the kidney cortex revealed activation of the NRF2 antioxidant response element signalling pathway (through upregulated JNK and MKK), although NRF2 expression itself did not significantly change. Oxidative stress may be one of the pathological mechanisms responsible for the deleterious effect of MGO in microvascular complications in the kidney, thus promoting vascular kidney damage.

The importance of oxidative stress in the progression of diabetic kidney disease has been noted in a few other transcriptomic studies. However, they mainly focus on glomeruli, using either STZ-induced diabetic mice [17] with marked hyperglycaemia or in vitro models [18]. A study by Morrison et al. reported upregulation of several thiol antioxidant genes in rat mesangial cells treated with high glucose concentrations [19].

The oxidative stress response signalling pathway underlies fibrosis and vascular changes during diabetic nephropathy progression as well as genes linked to inflammation and angiogenesis.

Canonical pathway enrichment showed the highest level of activation for the TGFβ signalling pathway, related to the development of renal fibrosis and nephropathy. TGFβ is a potent profibrotic cytokine present in several intracellular signalling pathways [20]. Our results pointed to MGO-induced activation of TGFβ pathway acting through the MAPK and Smad cascades in the kidneys of HHTg rats. TGFβ in kidney induces the production and secretion of extracellular matrix proteins [21].

TGFβ can also activate pro-inflammatory status, particularly in the presence of IL-6 [21] which may contribute to the progression of nephropathy. TGFβ accumulation in the kidney can play a critical role in elevating levels of microalbuminuria after MGO administration, by which TGFβ1 enhances glomerular permeability and attenuates tubular reabsorption of albumin. According to a recent meta-analysis [22], patients with T2D and nephropathy (*n* = 1,604) exhibited increased serum and urine TGFβ1 levels.

The TGFβ pathway represents a possible therapeutic target for renal fibrogenesis. Although TGFβ exerts fibrogenic and hypertrophic effects, doubt remains as to whether its suppression can prevent or treat nephropathy. Low TGFβ expression results in primary aldosteronism [23], while TGFβ deficiency in mice exacerbates the inflammatory response and tissue necrosis. Modulating GLO1 activity/expression represents another promising therapeutic approach. It has been shown that overexpression of GLO1 in *Glo1* transgenic mice prevented diabetes-induced MGO modification of glomerular proteins, increased oxidative stress, and the development of diabetic kidney disorders [24].

Transcriptome studies of human kidney biopsies in patients with diabetic nephropathy have reported changes in gene expression profiles linked to inflammation and angiogenesis [25]. In our study, angiogenesis in the kidney at the transcription level was inhibited by upregulation of the signalling pathway through thrombospondin 1, an endogenous inhibitor of angiogenesis that also influences the structure of the extracellular matrix. TSP1 inhibits angiogenesis directly by inducing endothelial cell apoptosis pathways (upregulated MAPK activates genes that lead to apoptosis), and indirectly activates TGFβ and inhibits VEGF-activated pathways. Thus, upregulation of this signalling pathway inhibits angiogenesis, stimulates apoptosis and activates TGFβ secretion. Physiological angiogenesis is impaired by MGO through RAGE-mediated and VEGFR2 degradation in cell cultures [26].

According to our transcriptomic analysis, MGO in the kidney also activates the inflammatory pathways that promote secretion of the pro-inflammatory cytokines IL-1, IL-6, IL-12 and TNFα.

The secretion of pro-inflammatory cytokines found in urine may represent an important diagnostic biomarker for early development of diabetic kidney disease, with urinary IL-6, IL-10 and TNFα secretion, in particular, reported to be the most sensitive cytokines for identifying renal disease in T2D patients [27].

Low chronic inflammation and abnormal production of pro-inflammatory cytokines are considered to play a significant role in a mechanism leading to the development and progression of diabetic kidney disease [28]. The inflammatory response is activated by metabolic and haemodynamic derangements in the diabetic kidney, occurring during the early stages of diabetes. Pro-inflammatory cytokines modify the renal structure and permeability of the glomerular endothelium, alter the expression of diverse molecules, increase ROS production, and can also induce apoptosis or cell necrosis. IL-6 affects extracellular matrix dynamics, stimulates the proliferation of mesangial cells, increases fibronectin expression, and enhances endothelial permeability [29].

Currently, the most reliable predictor of early kidney damage remains microalbuminuria. However, its predictive ability is limited in that not all diabetic patients with nephropathy exhibit increased levels of urinary albumin [30].

With respect to stages of the disease and risk of progression, proteomic-based analyses reveal that the combination of several biomarkers may be a more accurate method, offering more specific diagnostic and prognostic potential for determining early development of kidney failure than individual markers such as microalbuminuria.

One study investigated CKD273, a proteome-based classifier in patients with type 2 diabetes. Its risk score composition (patients at high and low risk) results documented changes in extracellular matrix components, including collagen fragments and tubular proteins, such as uromodulin, all early features of the disease [31].

Toward identifying a panel of biomarkers capable of predicting the progression of kidney failure in its early phases, a number of proteomic urine analyses of diabetic patients have been published. In uncomplicated diabetes, one such proteomic analysis demonstrated that early activation of fibrotic pathways in the kidney occurs before the onset of microalbuminuria [32].

Some recent studies have earmarked the urinary secretion of IL-6 [27] and epidermal growth factor (EGF) [33] as useful sensitive markers. Urinary secretion of EGF as a marker of tubular mass in a cohort from the Edinburgh Type 2 Diabetes Study was recently demonstrated to predict the decline of renal function independently of traditional risk factors and before the onset of microalbuminuria [34].

In contrast to blood, the urinary proteome is relatively stable. Urinary proteins mainly originate from kidney tissue (70%), with the remaining 30% deriving from plasma. Therefore, urine is the most reliable biomarker of renal disease and the vascular system. Proteomic studies suggest that MGO-induced changes in glomerular permeability and tubular reabsorption account for the proteins that appear in urine.

In our study of methylglyoxal administration, we observed activation of the profibrotic signalling pathway, the oxidative stress response, and pro-inflammatory pathways. Urinary proteomic and metabolomic markers reliably reflected transcriptomic signalling in the kidney, which is associated with MGO-induced kidney damage.

Although some studies [30, 35] provide evidence that MGO acts as an important signal molecule and affects different signalling pathways mainly involved in vascular pathophysiology, to our best knowledge this is the first study on the effect of MGO to combine transcriptome analysis of the kidney with urinary proteomic and metabolomic biomarker profiling.

Our transcriptome analysis shows that in order to identify molecular targets, a multi-pronged strategy must be adopted to ensure successful prevention and pharmacological intervention. In addition to normalising hyperglycaemia, the MGO/GLO1 balance must be properly managed during vascular complications in the kidney.

## Conclusion

Our results endorse the hypothesis that dicarbonyl stress plays a key role in renal microvascular complications, and are consistent at transcriptomic, proteomic and metabolomic levels. In the kidney, MGO not only activated profibrotic, proinflammatory pathways and the oxidative stress response, but inhibited angiogenesis at the transcriptomic level. These effects were further supported by urinary proteomic and metabolomic analysis, which reflected the kidney gene expression changes we observed. Our approach may be potentially useful in identifying diabetic kidney disease at an early stage, monitoring the progression, and novel therapeutic strategies.

## Data Availability

The datasets used during the present study are available from the corresponding author upon reasonable request.

## Abbreviations

AGEs: Advanced glycation end products
BW: Body weight
CML: Carboxymethyl lysine
FFA: Free fatty acids
GLO1: Glyoxalase 1
GSH: Reduced form of glutathione
GSSG: Oxidised form of glutathione
MCP-1: Monocyte chemoattractant protein-1
MDA: Malondialdehyde
MGO: Methylglyoxal
NRF2: Nuclear factor-erythroid 2-related factor-2
T2D: Type 2 diabetes
TG: Triglycerides
TGF-β: Transforming growth factor β
TNF-α: Tumor necrosis factor α
